# ATP synthase inhibitory factor subunit 1 regulates islet β-cell function via repression of mitochondrial homeostasis

**DOI:** 10.1038/s41374-021-00670-x

**Published:** 2021-10-04

**Authors:** Kailiang Zhang, Rong Bao, Fengyuan Huang, Kevin Yang, Yishu Ding, Lothar Lauterboeck, Masasuke Yoshida, Qinqiang Long, Qinglin Yang

**Affiliations:** 1 Division of Cardiology, Department of Internal Medicine, Tongji Hospital, Tongji Medical College, Huazhong University of Science and Technology, Wuhan, China.; 2 Cardiovascular Center of Excellence and Department of Pharmacology, Louisiana State University Health Science Center New Orleans, New Orleans, LA, USA.; 3 Department of Nutrition Science, University of Alabama at Birmingham, Birmingham, AL, USA.; 4 Department of Molecular Bioscience, Kyoto Sangyo University, Kamigamo-Motoyama, Kyoto, Japan.; 5 Guangdong Metabolic Diseases Research Center of Integrated Chinese and Western Medicine, Institute of Chinese Medicine, Guangdong Pharmaceutical University, Guangzhou, China.; 6 These authors contributed equally: Kailiang Zhang, Rong Bao.

## Abstract

Mitochondrial homeostasis is crucial for the function of pancreatic β-cells. ATP synthase inhibitory factor subunit 1 (IF1) is a mitochondrial protein interacting with ATP synthase to inhibit its enzyme activity. IF1 may also play a role in maintaining ATP synthase oligomerization and mitochondrial inner membrane formation. A recent study confirmed IF1 expresses in β-cells. IF1 knockdown in cultured INS-1E β-cells enhances glucose-induced insulin release. However, the role of IF1 in islet β-cells remains little known. The present study investigates islets freshly isolated from mouse lines with global IF1 knockout (IF1^−/−^) and overexpression (OE). The glucose-stimulated insulin secretion was increased in islets from IF1^−/−^ mice but decreased in islets from IF1 OE mice. Transmitted Electronic Microscopic assessment of isolated islets revealed that the number of matured insulin granules (with dense core) was relatively higher in IF1^−/−^, but fewer in IF1 OE islets than those of controlled islets. The mitochondrial ultrastructure within β-cells of IF1 overexpressed islets was comparable with those of wild-type mice, whereas those in IF1^−/−^ β-cells showed increased mitochondrial mass. Mitochondrial network analysis in cultured INS-1 β-cells showed a similar pattern with an increased mitochondrial network in IF1 knockdown cells. IF1 overexpressed INS-1 β-cells showed a compromised rate of mitochondrial oxidative phosphorylation with attenuated cellular ATP content. In contrast, INS-1 cells with IF1 knockdown showed markedly increased cellular respiration with improved ATP production. These results support that IF1 is a negative regulator of insulin production and secretion via inhibiting mitochondrial mass and respiration in β-cells. Therefore, inhibiting IF1 to improve β-cell function in patients can be a novel therapeutic strategy to treat diabetes.

## INTRODUCTION

ATP synthase is a protein complex that catalyzes and hydrolyzes ATP by utilizing the proton-motive force (PMF) from mitochondrial respiration and cellular energetics. ATP provision and mitochondrial function are essential for insulin production and secretion in the pancreatic β-cells^1^.

ATP synthase inhibitory factor subunit 1 (IF1) is an evolutionarily conserved mitochondrial protein interacting with the catalytic F1 sector of the mitochondrial ATP synthase and preventing ATP hydrolysis when PMF declines^2-7^. Multiple later studies suggest IF1 may also inhibit ATP synthesis, but IF1 protein inhibits proton translocation from the Fo to the F1 side with relatively lower efficiency^8^. Emerging evidence indirectly supports that IF1 inhibits oxidative phosphorylation (OXPHOS) and induces a metabolic switch toward glycolysis. A protein structure study using cryo-electron microscopy (Cryo-EM) demonstrates that IF1 dimer binds and inserts into the F1 heads of the ATP synthase tetramer to form the putative IF1-inhibited state of ATP synthase tetrameric structure^9^. Post-translational modifications of IF1 also contribute to IF1 activity alterations. IF1’s capacity to bind and inhibit ATP synthase can be inactivated via phosphorylation by a mitochondrial cyclic adenosine monophosphate-dependent protein kinase^10,11^.

A recent study suggests that IF1 may play a role in facilitating mitophagy^12-14^ and maintaining mitochondrial inner membrane structure^15^. Dysregulation of IF1 expression in different disease conditions has been reported^16-18^. To address this, in vivo studies using transgenic and gene targeting approaches have increasingly been used. Transgenic mice expressing an IF1 mutant with high-affinity binding to the F1 sector of ATP synthase were protective against neurotoxicity via a preconditioning effect of oxidative stress^19^. On the other hand, abolishing IF1 is protective against hepatocytotoxicity and in hearts subjected to mechanical stress^20,21^. Nevertheless, the mechanistic implication of different IF1 levels in various tissues remains incompletely understood.

Recent studies confirmed IF1 expresses in β-cells. In cultured INS-1E cells, glucose-induced insulin release was elevated in cultured INS-1E cells with IF1 knockdown^22^ and decreased with IF1 overexpression (OE)^23^. However, it remains unclear if this observation could be recapitulated in animals and the in-depth mechanisms underlying the effect of IF1 on insulin secretion in β-cells required further investigation. In the present study, we first investigate glucose-stimulated insulin secretion from islets of the IF1 knockout (IF1^−/−^) and the IF1 OE mice. We further determine how IF1 may affect β-cell mass, mitochondria morphology, and insulin storage in β-cells. In addition, we explore the biochemical effects of IF1 on cellular energetic changes using both gain and loss-of-function approaches in a β-cell line. Our findings demonstrate that IF1 represses mitochondrial energetics, leading to impaired β-cell function and insulin release. Silencing IF1 may be a therapeutic target to improve insulin secretion via upregulating cellular respiration and energy production.

## MATERIAL AND METHODS

### Animals

IF1^−/−^ Mice: Details of the IF1^−/−^ mice (homozygous IF1 knockout mouse line) were reported previously^15,21,24^. The mouse strain has been maintained in the C57/B6J background.

IF1 OE Mice: A transgenic mouse line (C57/B6J) with global IF1 OE was established using a previously described approach^25,26^. Briefly, the loxP-flanked chloramphenicol acetyltransferase gene is expressed under the control of the cytomegalovirus early enhancer/chicken β actin promoter, which drives ubiquitous transgene expression. The transgenes are silent without the removal of the chloramphenicol acetyltransferase gene. We crossed this line with the Ella-Cre mouse line with the ubiquitously expressed Cre recombinase at the embryonic state. The Cre-mediated recombination leads to the deletion of the chloramphenicol acetyltransferase gene and the global IF1 OE.

Animals received food and water on an ad libitum basis, and lighting was maintained on a 12-h cycle. All experimental procedures were conducted in accordance with the Guide for Care and Use of Laboratory Animals of the National Institutes of Health, and were approved by the Institutional Animal Care and Use Committee of the University of Alabama at Birmingham and Louisiana State University Health Science Center-New Orleans.

### Quantitative real-time polymerase chain reaction (PCR)

Total RNA samples were isolated from islets and INS-1 cells using an RNA extraction kit (Zymo) according to the manufacturer’s instructions. Quantitative real-time PCR analyses were carried out using the Step One Real-time PCR system (Applied Biosystems) to determine transcript levels of target genes. Expression of each gene was normalized to β-actin or 36B4 and compared across conditions.

### Islet isolation

Islets were isolated from IF1^−/−^, IF1 OE, and control mice at the age of 10 weeks. After dissection, Clzyme-RI (Vitacyte, Indianapolis, IN) was injected into the pancreas through the bile duct ampullae, and pancreas samples were collected and digested for 17 min at 37 °C with shaking every 5 min^27,28^. Subsequently, ice-cold Hank’s balanced salt solution (HBSS) (Thermo Fisher Scientific, #24020117) was added and shaken until the pancreas-HBSS solution became homogenous. Samples were centrifuged at 1500 × *g* for 2 min at 4 °C. Pellets were resuspended with HBSS, filtered through a 400 μm filter mesh, and centrifuged at 1500 × *g* for 2 min at 4 °C. Pellets were then resuspended in Histopaque 1119 (Sigma-Aldrich), Histopaque 1077 (Sigma-Aldrich), and HBSS, which were sequentially added to make a 3-layer solution in a falcon tube. After centrifugation at 14,000 × *g* for 30 min at room temperature, monolayered islet cells were collected from the middle layer containing Histopaque 1077 and washed twice with ice-cold HBSS.

### Glucose-stimulated insulin secretion (GSIS)

The GSIS assay was conducted as described previously^29^. Briefly, islets freshly isolated from mice were cultured in 24-well plates and preincubated in Krebs–Ringer bicarbonate (KRB) solution containing 3 mmol/l glucose for 30 min at 37 °C. Then, the supernatant was replaced with 400 μl of the above KRB solution and incubated for 1 h at 37 °C. Following plate centrifugation (1000 rpm, 5 min), the supernatants were collected (marked as low glucose). Next, a fresh KRB solution containing 17 mmol/l glucose was added at 400 μl per well. Following another hour of incubation at 37 °C, the supernatants were collected. Insulin concentrations in the supernatants were measured using insulin ELISA kits following the instructions (ALPCO Diagnostics, Salem, NH). For insulin measurement in islets, freshly isolated islets were homogenized in acid-ethanol solution (0.2 M HCl in ethanol) at 4 °C overnight. The supernatant was collected by centrifugation at 4000 rpm for 20 min at 4 °C. The level of insulin was measured using the same ELISA kits as above, following the manufacturer’s instructions.

### Ultrastructural assessments of islets

Isolated islets were collected and fixed with fixation solution (2% v/v paraformaldehyde, 2.5% v/v glutaraldehyde, and 0.1 M sodium cacodylate [pH 7.3]) at room temperature overnight. The ultrastructure of islets was examined using a Tecnai Spirit T12 electron microscope (Thermo-Fisher).

### Immunohistochemistry

The fixed pancreas was embedded in the Tissue Tek optimal cutting temperature compound under dry ice and sectioned immediately in a freezing cryostat at −20 °C for immunofluorescence analysis. The blocks were cut into 5 μm sections, fixed with 100% Methanol for at least 30 s and dried for 30 min at room temperature. After that, sections were washed with phosphate-buffered saline (PBS). The slides were later incubated for 10 min with 3% hydrogen peroxide to quench the endogenous peroxidase activity. Following blocking with 5% normal horse serum in PBS, the sections were incubated overnight at 4 °C with specific primary antibodies (Insulin H-86; Santa Cruz Biotechnology). Slides were washed and covered by secondary antibodies (DyLight 594 Goat Anti-Rabbit Immunoglobulin G) for incubation with a light block at room temperature in a humidity chamber for 60 min. The sections were finally embedded in ProLong® Gold Antifade Mountant with 6-diamidino-2-phenylindole (Thermo).

Fluorescence immunocytochemistry was performed to determine IF1 protein expression in the INS-1 cells. Briefly, cells were fixed by 4% formaldehyde for 15 min at room temperature, washed three times with PBS, blocked with 5% bovine serum albumin, and incubated for 1 h at room temperature. After blocking, cells were incubated with primary antibody IF1 (12067-1-AP, Proteintech) overnight at 4 °C, and incubated with cy3 fluorescent secondary antibody (GB21303, Servicebio) for 2 h at room temperature in the dark. DAPI was used for nucleus staining.

### β-cell mass measurement

μ-cell mass in sections was determined by thresholding and quantitating insulin-positive areas in nonadjacent sections at 100-μm intervals throughout the pancreas. All images were captured using FLoid Cell Imaging Station-Demo Video (Invitrogen) and a Cytation 5 Cell Imaging Multi-Mode Reader (BioTek). Color deconvolution was based on the orthonormal transformation of the original RGB images. For each cohort, we analyzed a minimum of 15 individual whole-pancreatic immunofluorescence slides. The image analysis was done using the ImageJ software. Quantification of β cell mass was performed as described previously^30^.

### Western blot analysis

We extracted total protein from isolated mouse islets and INS-1 cells and determined the protein concentration by bicinchoninic acid assay. Protein was prepared with sample loading buffer (Recipe from Cold Spring Harbor Protocols (http://cshprotocols.cshlp.org/content/2015/12/pdb.rec087791.full?text_only=true)) and boiled. A constant amount of protein was loaded and separated by sodium dodecyl sulfate-polyacrylamide-gel electrophoresis. Protein was transferred onto polyvinylidene fluoride membranes, and immunoblot analyses were carried out using antibodies from commercial sources, following the manufacturer’s instructions. Antibody for immunoblotting included IF1 (12067-1-AP Proteintech and Ab110277, Abcam), ATP5B (Abcam, ab14730), ATP5G (Abcam, ab96655), tubulin (Cell signaling #2144) and GAPDH (A01020, Abbkine). HRP-conjugated secondary antibodies were purchased from Santa Cruz Biotechnology and Boster Bio. The immunoblotting images were captured with Tanon-5200 chemiluminescent imaging system (Tanon) by developing the membranes in Enhanced ECL Chemiluminescent Substrates (36222ES60, Yease).

### Cell culture, transfections, and siRNA-mediated IF1 knockdown

Rat insulinoma INS-1 cells (Lonza Corporation) were cultured in RPMI 1640 medium (PYG0006, Boster) containing 2000 mg/L D-glucose, 300 mg/L glutamine, 2000 mg/L sodium bicarbonate, supplemented with 15% (v/v) fetal calf serum, 50 μM β-mercaptoethanol, 50 IU/ml penicillin, and 50 μg/ml streptomycin. Cells were incubated in humidified air with 5% CO_2_ at 37 °C. Adenovirus with IF1 (NM_016311) for IF1 OE (Ad-IF1) was generated via service contract (VigeneBio). Ad-IF1 (50 MOI) was added to cultured INS-1 at 70–75% confluence. Fresh media were added to replace the viral media after two hours. After 48 h of culture, cells were collected for immunoblot or used for XFe assay. For IF1 knockdown, two siRNA duplexes against Rattus Norvegicus IF1: [5′-CTGAGCAACGCCGAAGATA-3′(sense)], [5′-TCGTCGGAGAGCATGGATT-3′ (sense)] with 70 nM concentration were used^22^. The transfection was performed using riboFECT™ CP reagent (Ribobio) according to the manufacturer’s instructions. Scrambled siRNA nonspecific to any Rattus Norvegicus gene (si-NC) was used as a control.

### Measurement of cellular respiration rates

Cellular oxygen consumption rates (OCR) were determined in an XFe 24 Extracellular Flux Analyzer (Agilent Seahorse Bioscience). After transfection, cells were trypsinized and seeded at 60,000 cells per well of cell culture microplates (Seahorse Bioscience) and incubated at 37 °C and 5% CO_2_ overnight. The XF Base Medium (103334-100) with substrates of 25 mM glucose, 0.5 mM pyruvate, and 2.5 mM L-glutamine was freshly prepared and pH adjusted to 7.4. OCR was measured after 2 μg/ml oligomycin (Oligo), 1 μM carbonyl cyanide 4-trifluoromethoxy-phenylhydrazone (FCCP), 4 μM Antimycin A (AA) and 1 μM rotenone (Rot) sequential injection. After the measurement, OCR was normalized to protein content.

### Mitochondrial membrane potential and imaging of mitochondrial structure and network

Mitochondrial membrane potential was analyzed using a Tetramethyl rhodamine methyl ester (TMRM)-mitochondrial membrane potential assay kit (CAS#: 115532-50-8, AAT Bioquest). Briefly, INS-1 cells were seeded into black 96-well plates (60 K cells/well). Cells were treated with 50 nM TMRM in the culture medium and incubated at 37 °C and 5% CO_2_ for 30 min. Cells were treated with oligomycin (2 μg/ml final concentration) as a positive control for the mitochondrial membrane potential assay. After the incubation, cells were washed twice with PBS and cultured in fresh media without TMRM. The intensity of TMRM fluorescence was read on a multimode fluorescence microplate reader at excitation/emission wavelengths of 549/573 nm.

Mitochondria in INS-1 cells were visualized by TMRM staining as described above, and imaging was conducted using the inverted laserscanning confocal microscope (Olympus FV1000) with a 60x oil immersion objective. The shape factors mean branch length aspect ratio (AR) and form factor (FF) were analyzed by ImageJ with the mitochondrial analyzer plugin to evaluate mitochondrial elongation and branching.

### ATP content assay

Relative ATP content in INS-1 cells was measured with the ATP assay kit (S0026, Beyotime Biotechnology) according to the manufacturer’s instructions. Briefly, after washing twice with PBS, cells were treated with lysis solution and incubated on ice for 15 min. After the incubation, cells were centrifuged (12,000 × *g*, 5 min at 4 °C), and the supernatant was reserved. ATP assay working solution and the supernatant were added into black 96-well plates with multichannel pipettes in sequence. The samples corresponding luminescence were measured in Synergy” 2 Multi-Detection Microplate Reader.

### Lactic acid assay

The extracellular lactate levels were measured using a lactate assay kit (A019-2, Nanjing Jiancheng Bioengineering) according to the manufacturer’s instructions. Briefly, INS-1 cells were treated with culture media containing 0.5% fetal bovine serum and incubated at 37 °C, 5% CO_2_. After 2 h, the medium containing lactic acid was collected and measured according to the manufacturer’s protocol. The values were normalized to total protein.

### Reactive oxygen species (ROS) assays

To determine potential ROS changes, we quantified the cultured INS-1 cells using DCFH-DA staining and MitoSOX Red assays. DCFH-DA was applied to detect the whole cell ROS level. DCFH-DA (S0033, Beyotime) was diluted 1:1000 in RPMI 1640 media without serum at a final concentration of 10 μM. After 30 min incubation, the cells were washed three times using PBS and fresh culture media was added. Similarly, MitoSOX Red (M36008, Invitrogen™), a mitochondrial superoxide indicator, was used to detect mitochondria-generated ROS. Briefly, INS-1 cells in black 96-well plates (60 K cells/well) were incubated with MitoSOX Red at a final concentration of 5 μM at 37 °C, 5% CO_2_ in the dark for 30 min and AA (4 μM final concentration) was incubated simultaneously with MitoSOX Red as the positive control. The fluorescence signal of DCFH-DA was excited by 485/20 nm following a bandpass filter of 528/20 nm. MitoSOX Red was evaluated by Multi-Detection Microplate Reader with 530/25 nm excitation and a bandpass filter of 590/35 nm.

### Statistical analyses

Data for 2-group comparisons were analyzed with the nonparametric two-tailed Student’s *t* test; otherwise, data were analyzed by one-factor or mixed, 2-factor ANOVA and multiple comparisons using the GraphPad Prism 8 software (GraphPad Software Inc.). Values of quantitative results were expressed as mean ± SD. Differences between groups and treatments were regarded as significant at *p* < 0.05.

## RESULTS

### Islets isolated from IF1^−/−^ mice show improved glucose-stimulated insulin release

**Fig. 1:**
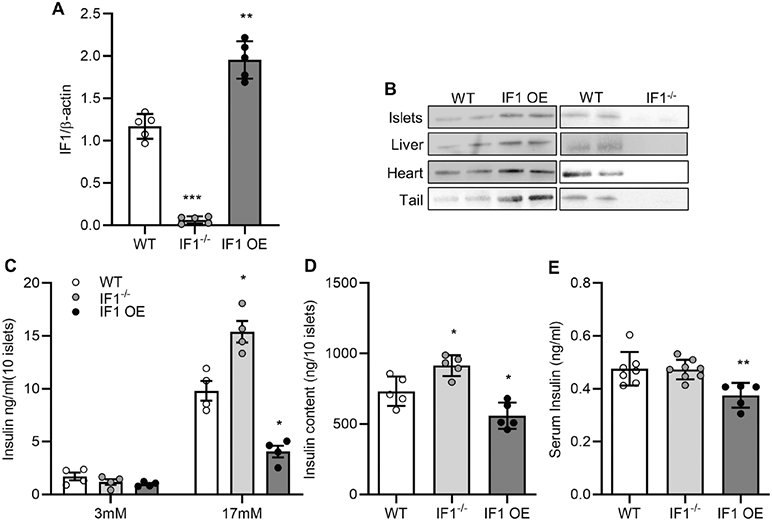
Effects of IF1 on insulin release in islets from mice. **A** Real-time PCR measurement of IF1 mRNA in islets from mice. **B** Western blots on samples from different tissues of IF1^−/−^, IF1 OE and WT mice. **C** Glucose-stimulated insulin release determined by GSIS assays from islets of IF1^−/−^, IF1 OE and WT mice. **D** ELISA measurement of insulin contents in isolated islets from IF1^−/−^, IF1 OE and WT mice. **E** ELISA measurement of serum insulin levels in IF1^−/−^, IF1 OE and WT mice after fasting for 6 h. Data are expressed as mean ± SD. *P <0.05, **P <0.01, ***P < 0.001 vs WT mice. Data were analyzed using one-way ANOVA followed by Dunnett’s test.

A recent study validates that IF1 is present in the mitochondria of pancreatic islets and IF1 silencing in cultured rat insulinoma INS-1E cells enhances glucose-stimulated insulin release^22^. However, the role of IF1 in islet β-cells from animals remains unknown. In addition to investigating the previously reported IF1^−/−^ mice, we have also generated a transgenic mouse line with global IF1 OE. Real-time PCR measurement of IF1 transcript on samples extracted from islets from IF1^−/−^ and IF1 OE confirmed the complete IF1 KO in islets from IF1^−/−^ mice (Fig. 1A) and about a 2-fold increase of IF1 in islets from the IF1 OE mice (Fig. 1A). Western blots confirmed the consistent protein expression in islets and other tissues, such as the heart, liver, and tail (Fig. 1B). Under basal conditions, the IF1^−/−^ and IF1 OE mice show normal growth with no overt phenotype at least up to ten months of age. Basal glucose contents were comparable among IF1^−/−^, IF1 OE, and wild-type (WT) mice.

To determine how IF1 determines islet response to high glucose stimulation, we isolated islets from IF1^−/−^ and IF1 OE mice and conducted the GSIS assay. GSIS assays revealed that insulin secretion was elevated in IF1^−/−^ but decreased in IF1 OE islets compared with WT controls when subjected to high glucose (17 mM) (Fig. 1C). On the other hand, islets treated with 3 mM glucose showed no difference (Fig. 1C). Interestingly, insulin content was higher in IF1^−/−^ but lower in IF1 OE islets than in WT (Fig. 1D). After 6 h of fasting, serum insulin content was similar between WT and IF1^−/−^ mice but significantly decreased in IF1 OE mice (Fig. 1E). These results, for the first time, validate that IF1 plays an inhibitory role in islet insulin release in mice.

### IF1 contents affect pancreatic β-cell mass in mice

**Fig. 2:**
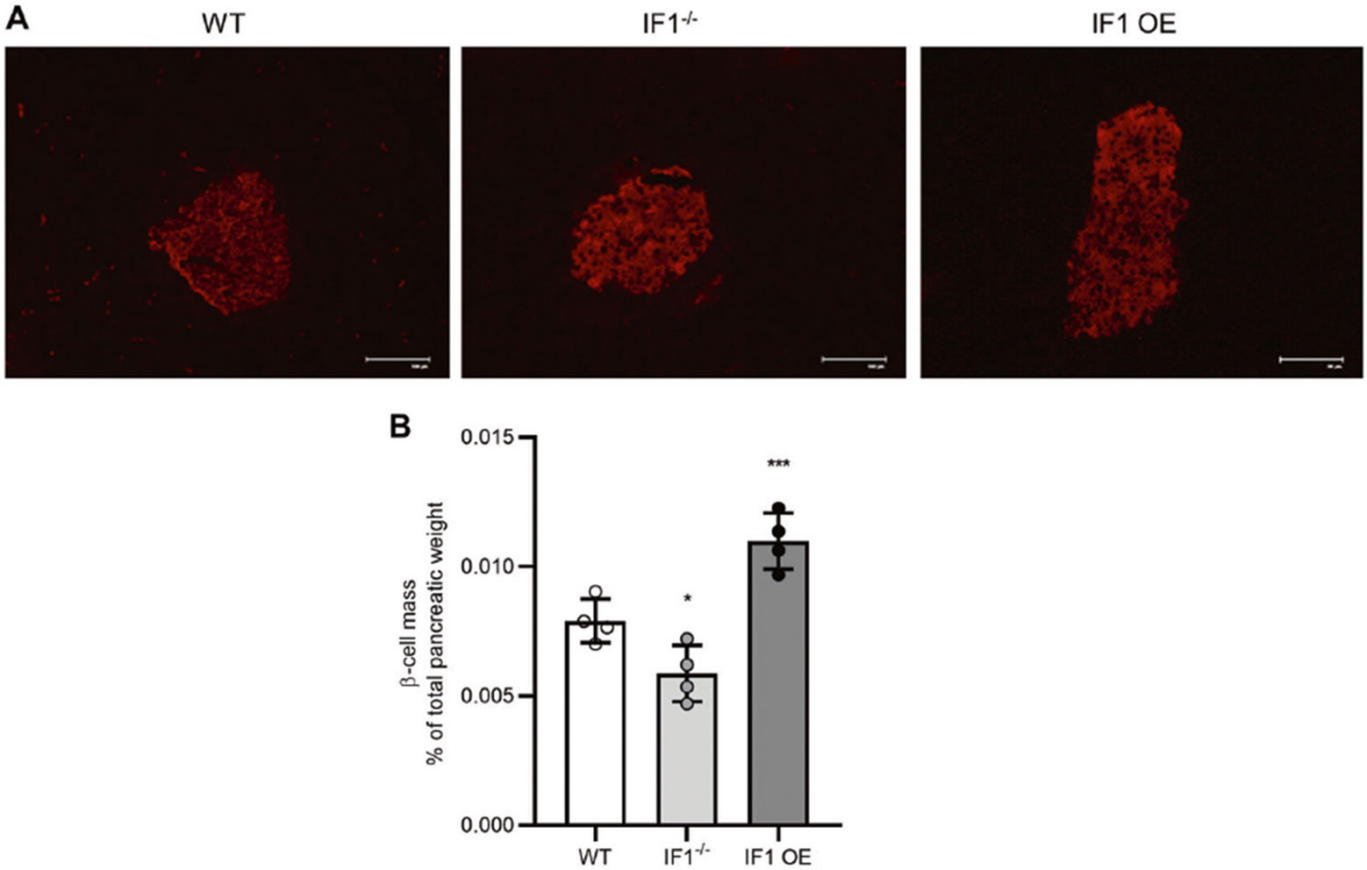
Effects of IF1 on β-cell mass in pancreas from mice. **A** Immunofluorescent staining of insulin on frozen section of pancreas from IF1^−/−^, IF1 OE and WT mice. **B** β-cell mass quantification. Data are expressed as mean ± SD. *P < 0.05, **P <0.01, ***P < 0.001 vs WT mice. Data were analyzed using one-way ANOVA followed by Dunnett’s test.

To investigate if β-cell mass may reflect the differences in glucose-stimulated insulin release from isolated islets, we measured β-cell mass in IF1^−/−^, IF1 OE and WT mice. Image analysis on fluorescent insulin staining (Fig. 2A) normalized to pancreatic weight revealed that β-cell mass was decreased in IF1^−/−^ and increased in IF1 OE compared with WT mice (Fig. 2B). Therefore, these results suggest that IF1 contents are inversely related to β-cell mass in response to insulin production and release demand.

### IF1 affects the insulin contents in insulin granules within β-cells

**Fig. 3:**
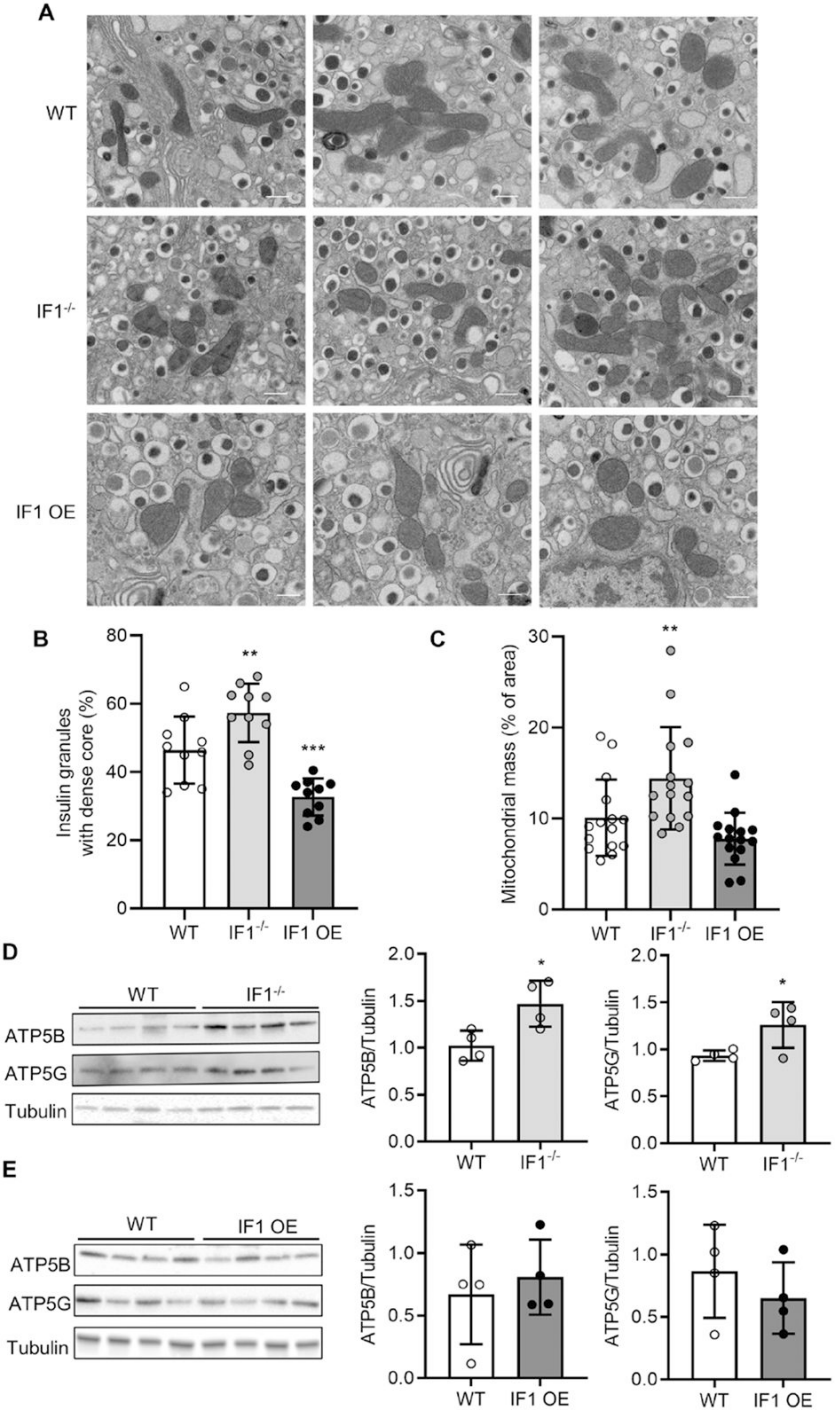
β-cell ultrastructural changes assessed by transmission EM on isolated islets from mice. **A** Electron micrograph of β-cells showing mitochondria and insulin-containing secretory granules. **B** Quantification of insulin granules with dense core relative to total granules’ number. **C** Quantification of mitochondrial mass relative to section area. Islets were isolated from 3 mice of each experimental group. **D** Western blots on total protein samples from islets of IF1^−/−^ and WT mice. **E** Western blots on total protein samples from islets of IF1 OE and WT mice. Data are expressed as mean±SD. *P < 0.05, **P < 0.01, ***P < 0.001 vs wild-type mice. Data were analyzed using one-way ANOVA followed by Dunnett’s test and Student’s *t* test.

To determine potential changes in insulin storage and release in islets from mice, we assessed the ultrastructure of isolated islets from IF1^−/−^, IF1 OE and WT mice using transmission EM. Imaging analyses revealed that the insulin granule size and number were largely similar among the three groups (Fig. 3A). β-cells within islets from IF1^−/−^ and IF1 OE mice were assessed on EM micrographs. Compared with WT β-cells, the ratios of insulin granules with dense core to the total granules were increased in IF1^−/−^ β-cells and decreased in IF1 OE β-cells (Fig. 3B). These results support that IF1 plays a role in insulin production and release from islet β-cells.

The mitochondria within β-cells of IF1 OE islets were comparable with those from WT mice, whereas those in IF1^−/−^ β-cells showed an increased mitochondrial mass (Fig. 3C). Furthermore, western blot on total protein samples from islets revealed that ATP5B and ATP5G, the two ATP subunits, were increased in IF1^−/−^ compared with that of WT islets (Fig. 3D), whereas there was no difference between the IF1 OE and controlled islets (Fig. 3E). These results suggest that IF1 may reduce insulin production and release due to its direct or indirect role in regulating mitochondrial mass within the islet β-cells.

### Effects of IF1 on mitochondrial structure and function in cultured INS-1 cells

**Fig. 4:**
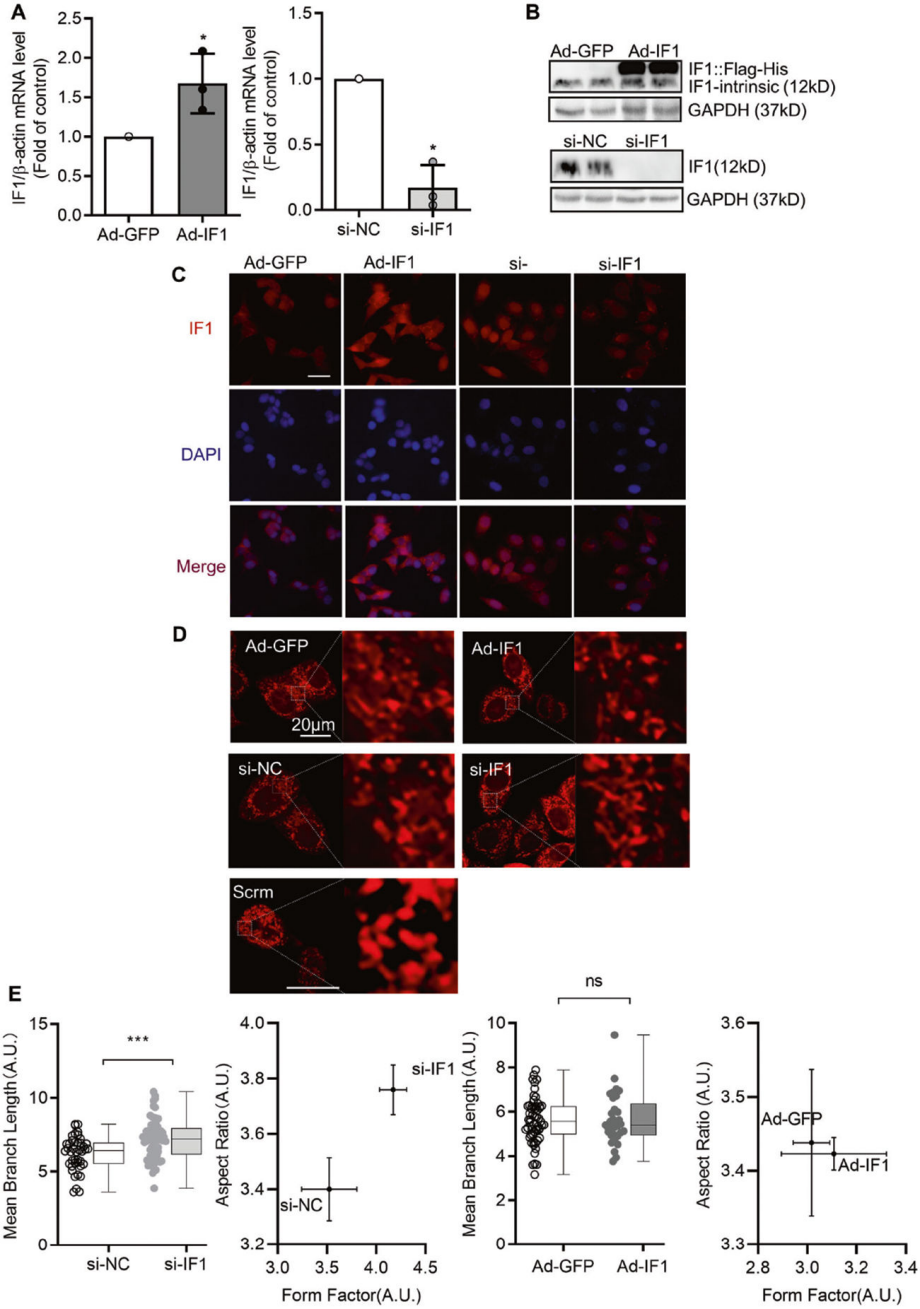
IF1 OE and knockdown in INS-1 cells. **A** Real-time PCR measurement of IF1 transcript on RNA samples extracted from cultured INS-1 cells with adenovirus-mediated IF1 OE and si-RNA- mediated knockdown. **B** Western blots on protein samples extracted from cultured INS-1 cells with adenovirus-mediated IF1 OE and si-RNA- mediated knockdown. **C** Images of immunofluorescent staining of IF1 on cultured INS-1 cells with adenovirus-mediated IF1 OE and si-RNA- mediated knockdown. **D** IF1 influences mitochondrial network morphology in cultured INS-1 cells. Representative images of mitochondrial network morphology in cultured INS-1 cells after IF1 OE and knockdown. **E** Mitochondrial network morphology analyzed by ImageJ with plugin Mitochondria Analyzer. Data are presented as mean ± SD; *n* = 3, *P < 0.05, **P < 0.01, ***P < 0.001 vs control. Data were analyzed using Student’s *t* test.

To further investigate IF1’s potential role in regulating β-cell function via regulating mitochondria, we assessed cellular bioenergetics in response to IF1 OE and knockdown in INS-1 cells, a cultured β-cell line. Real-time PCR results support the corresponding IF1 level changes after transfection of adenovirus-IF1 and siRNA-IF1 (Fig. 4A). Western blots and immunostaining confirmed the effects of adenovirus-mediated IF1 OE and si-RNA-mediated IF1 knockdown in the INS-1 cells (Fig. 4B, C). Additionally, we examined the INS-1 cells using a confocal microscope on their structure upon TMRM staining (Fig. 4D). We first assessed the impact of IF1 on mitochondrial network change after IF1 OE and knockdown. The mitochondrial network became more elongated (aspect ratio) and branching (form factor) in IF1 knockdown but had no overt changes in IF1 OE cells (Fig. 4D, E). This increased mitochondrial network is consistent with the finding that mitochondrial mass is increased in IF1^−/−^ islets.

**Fig. 5:**
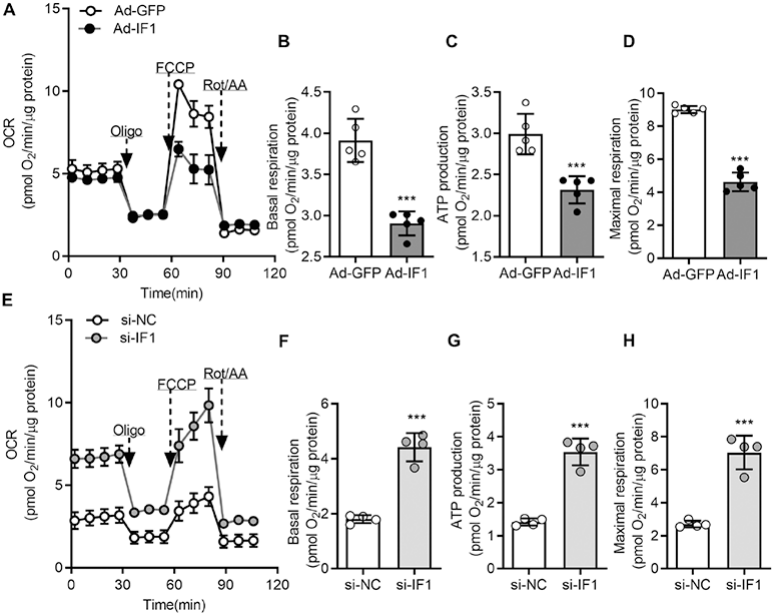
Effects of IF1 on mitochondrial oxidative phosphorylation. **A** INS-1 cells were transfected with adenovirus. The respiratory profile was measured by Seahorse XFe analyzer (mean ± SD *n* = 5 in each group). **B** The basal respiratory rates were measured after INS-1 cells were transfected with adenovirus. Data are presented as mean ± SD; *n* = 5 in each group. **C** Mitochondrial ATP production-related respiratory rates were measured in INS-1 cells after adenovirus transfection. Data are presented as mean ± SD; *n* = 5 in each group. **D** Mitochondrial maximum respiratory rates were measured in INS-1 cells after adenovirus transfection (mean ± SD *n* = 5 in each group). **E** INS-1 cells were transfected with siRNA-NC or siRNA-IF1. The respiratory profile was measured by Seahorse XFe analyzer (mean ± SD *n* = 4 in each group). **F** The basal respiratory rates were measured after INS-1 cells transfected with siRNA-NC or siRNA-IF1. Data are presented as mean ± SD; *n* = 4 in each group. **G** Mitochondrial ATP production-related respiratory rates were measured after injection of oligo in INS-1 cells after siRNA-NC or siRNA-IF1 transfection. Data are presented as mean ± SD; *n* = 4 in each group. **F** Mitochondrial maximum respiratory rates were measured in INS-1 cells after siRNA transfection in response to FCCP and confirmed by Rot/AA treatment. Data are presented as mean ± SD, *n* = 4. ***P < 0.001. Data were analyzed using Student’s *t* test.

**Fig. 6:**
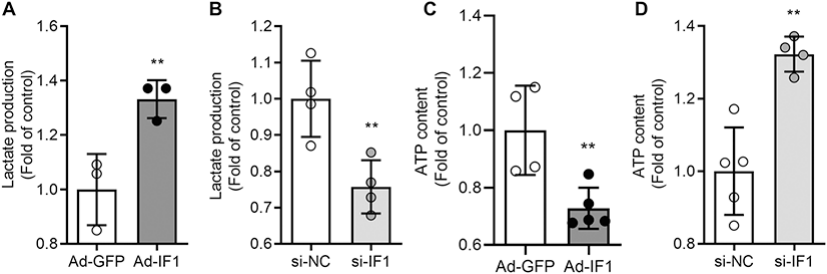
Effects of IF1 on ATP content and glycolysis flux in cultured INS-1 cells. **A** Lactate release rate was determined after adenovirus transfection in INS-1 cells. Data are presented as mean ± SD; *n* = 3 independent experiments. **B** Lactate release rate was determined after siRNA transfection in INS-1 cells (mean ± SD; *n* = 4 independent experiments). Oligo was added as positive control to inhibit mitochondrial oxidative phosphorylation (mean ± SD; *n* = 3 independent experiments). **C**, **D** ATP content in INS-1 cells was determined after adenovirus or siRNA transfection. (4–5 independent experiment samples in each group; error bars denote mean ± SD). **P < 0.01 vs control. Data are expressed as mean ± SD. Student’s *t* test is used.

Cellular respiration was determined using the Seahorse Bioanalyzer after adenovirus and si-RNA transfection in INS-1 cells. We first confirmed that IF1 OE or knockdown did not overtly change cellular proliferation and survival for the during experiments. The exact number (60,000/well) of INS-1 cells were seeded and cultured to measure cellular bioenergetics. We measured oxygen consumption rate (OCR) before and after adding oligomycin (Oligo) to block ATP synthase complex activity, FCCP to uncouple respiration, and ROT and AA to inhibit complex I and III activities. IF1 OE significantly repressed the basal and maximal respiration rates and ATP production rates (Fig. 5A-D). We then determined the alteration of mitochondrial respiration function after IF1 knockdown. INS-1 cells with IF1 knockdown exhibited robustly increased basal and maximal respiration rates along with ATP production rates (Fig. 5E-H). Corresponding with mitochondrial respiration function, IF1 OE increased and IF1 knockdown decreased lactic acid production rates (Fig. 6A, B). Direct measurements of ATP contents in these cells revealed consistent changes of ATP upon changes of IF1, in which IF1 OE reduced and IF1 knockdown raised cellular ATP contents (Fig. 6C, D). These results suggest that IF1 is a negative regulator of mitochondrial respiration in β-cells with reduced energy production.

### IF1 regulates ROS level and mitochondrial membrane potential

**Fig. 7:**
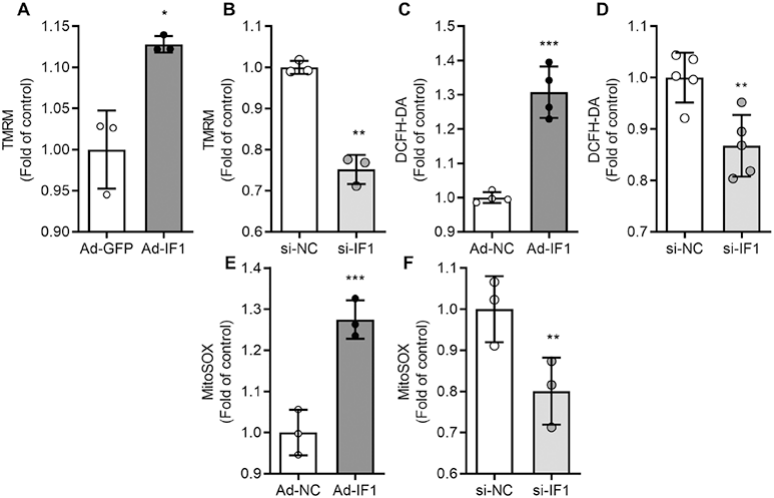
IF1 influences mitochondrial membrane potential and ROS level in cultured INS-1 cells. **A**, **B** Mitochondrial membrane potential was determined by TMRM treatment, and the corresponding fluorescence signal intensity was recorded and calculated. (*n* = 3 independent experiments). **C**, **D** The whole-cell ROS level in INS-1 cells was determined after IF1 OE (*n* = 4 independent experiments) and knockdown (*n* = 5 independent experiments). DCFH-DA fluorescence signal intensity was measured and calculated. **E**, **F** The mitochondrial ROS level in INS-1 cells was determined after IF1 OE or knockdown. mitoSOX fluorescence signal intensity was measured and calculated. (*n* = 3 independent experiments). *P < 0.05, **P < 0.01, ***P < 0.001 vs control. Data are expressed as mean ± SD. Data were analyzed using Student’s *t* test.

To determine if the cellular energetic inhibitory effects of IF1 may lead to changes in mitochondrial membrane potential and ROS production, we assessed mitochondria membrane potential after IF1 knockdown and OE in cultured INS-1 cells. We found that IF1 knockdown reduced and IF1 OE increased mitochondria membrane potential (Fig. 7A, B). Furthermore, assays with DCFH-DA for whole cells ROS production revealed that IF1 OE produced more and IF1 knockdown produced less ROS than controls in cultured INS-1 cells (Fig. 7C, D). Similarly, mitochondrial superoxide content indicated by the mitoSOX signal was elevated in IF1 OE and declined in IF1 knockdown INS-1 β-cells (Fig. 7E, F). Therefore, these results provide additional evidence supporting that IF1 is a negative regulator of mitochondrial respiration and energy metabolism in β-cells.

## DISCUSSION

Impaired insulin production and secretion from the pancreatic β-cells is a critical event in diabetes^31^. The β-cell mitochondria are the primary source of energy for the exocytosis of insulin-containing vesicles. ATP plays a crucial role in regulating glucose-stimulated insulin secretion by controlling the closure of ATP-sensitive potassium channels, subsequent plasma membrane depolarization, and activation of voltage-gated L-type Ca^2+^ -channels^32,33^. In the present study, we provide evidence to support a critical role of IF1, an ATP synthase inhibitory protein, in regulating islet β-cell function and insulin release via regulating ATP synthesis and mitochondrial homeostasis.

ATP synthase is a molecular machine in the final phase of OXPHOS that produces most cellular ATP. ATP synthase can be a key determinant of insulin secretion. ATP synthase inhibited by oligomycin leads to reduced GSIS in β-cells^34^. Changes in ATP synthase β-subunit expression are directly correlated to cellular ATP levels in INS-1 cells^35,36^. In female BHD/cdb rats with mutated ATP synthase, β-cell insulin secretion is impaired^37^. However, it remains under-studied regarding the specific effects of ATP synthase on determining β-cell function and insulin production/release.

IF1 interacts with the F1 sector when PMF collapses under pathological conditions^2,6-8^. A recent cryo-electron microscopy study indicates that the two IF1 in antiparallel arrangement binds with two V-shaped mammalian ATP synthases to form the H-shaped tetramer, an inhibitory state of ATP synthase^9^. Although most early studies revealed that IF1 inhibits the reversed ATP synthase activity to reduce ATP hydrolysis, later studies on insideout sub-mitochondrial vesicles suggest that IF1 also inhibits the forward proton translocation (from the Fo to F1 side) with less efficiency^8^. Transgenic OE of an IF1 H49K mutant spontaneously binds with ATP synthase F1 sector to suppress ATP synthase activity, leading to a glycolytic shift^16,19,38,39^. Bi-directional-ATP synthase activities are reduced in colonocytes of the intestine-specific IF1 OE mice^40^. Recent studies prove that endogenous IF1 in cultured INS-1E β-cells inhibit ATP synthesis and cellular respiration, which inversely regulates insulin secretion^22,23^. Our results support IF1’s negative regulatory effect on cellular OXPHOS rate in β-cells. In cells with active OXPHOS, such as β-cells, IF1’s negative ATP synthase regulation appears to play a role in preventing overactive OXPHOS at basal conditions.

The physiological function of IF1 in different tissues in animals remains incompletely understood. IF1 deletion in mice does not lead to overt phenotype from development to adulthood^24^. Still, these mice are protected from pathological cardiac hypertrophy induced by pressure overload, probably via conserving mitochondrial function and ATP content^21^. Another IF1 knockout mouse line showed improved hepatocyte survival to AA-induced cytotoxicity^20^. On the other hand, mice with neuron-specific transgenic OE of the IF1 H49K mutant were protected from neurotoxins via a ROS-induced preconditioning mechanism derived from IF1’s OXPHOS inhibitory effects^19^. Similarly, inhibition of ATP synthase activity in colonocytes in mice overexpressing IF1 protects the colon from dextran sodium sulfate-induced colitis via ROS-activated inflammation that recruits M2-macrophages^40^. Moreover, ATP synthase reacts quickly and reversibly to metabolic conditions, not only by functional but also by spatial and structural reorganization. In IF1-knockdown cells, ATP synthase was more mobile^41^, which may also improve the energetic state. Therefore, while IF1 may exert differential effects across different tissues, IF1 appears to exert an inverse regulatory role in ATP and subsequently β-cell insulin storage and release.

Mitochondrial energetics is a valuable predictor of islet quality and long-term nutrient responsiveness^42^. Gene mutations that impair mitochondria function can induce islet dysfunction and lead to diabetes^43,44^. Our present findings demonstrate that IF1 represses mitochondrial respiration, probably due to its inhibitory effect on ATP synthase. In contrast, inactivating IF1 reverses and enhances mitochondrial respiration, thus facilitating islet β-cell function with more insulin storage and release in response to glucose.

There is a subtle balance between β-cell insulin secretion and β-cell mass under physiological conditions. Recent transcriptome analysis indicates hundreds of proliferative genes, but no glucose sensing and insulin secretion genes were upregulated in mouse proliferating β cells^45^. Meanwhile, increased insulin production after islet transplantation reduces β-cell replication in the host pancreas^46^. In our study, IF1 knockout decreased, and IF1 OE increased islets β-cell mass, probably because IF1 knockout increased β-cell OXPHOS rate and insulin secretion and vice versa. However, further investigations are required to determine if β-cell proliferation was also altered in response to IF1 changes.

A recent report showed that IF1 is present in β-cells and binds to the ATP synthase even under normal physiological conditions^22^. Although we did not detect overt phenotype in both the IF1^−/−^ and IF1 OE mice, we did detect relatively lower serum insulin in IF1 OE mice. Moreover, the observed ultrastructural changes in islet β-cells support that IF1 exerts its inhibitory effects on the mitochondria even under physiological conditions. The chronic inhibitory effects of IF1 may lead to defective insulin storage and release. Upon acute stimulation with high glucose, the effects of IF1 become markedly manifested. One limitation in the current study is that the gain- and loss-of-function mouse models of IF1 were systemic. Potential confounding effects of IF1 on other metabolic tissues, such as skeletal muscle^47,48^, liver^15,20^, intestine^40^, brain, and the heart^10,21^, prevent further assessment of the in vivo response of islet under hyperglycemic conditions. However, investigations to determine how these mice respond to obesity and diabetes should be highly informative.

The analysis of IF1 function under the physiological condition in cultured INS-1 β-cells showed that IF1 OE led to reduced ATP and increased mitochondrial membrane potential, whereas IF1 knockdown increased ATP and decreased mitochondrial membrane potential. These findings are consistent with the recent reports in β cell and tumor cell lines^16,22^. However, it appears that the IF1 may exert its effects differently in different tissues. In differentiating mouse erythroleukemia cells, IF1 knockdown leads to cellular ATP depletion and mitochondrial membrane potential hyperpolarization^49^. IF1 facilitates mitochondrial cristae formation in Hela and hepatocytes^15^, but we did not observe similar changes in IF1^−/−^ and IF1 OE islets. Instead, EM images of mitochondria in islet sections exhibited a modestly increased and decreased mitochondrial mass, respectively. The high ATP5B and ATP5G protein levels on IF1 knockout islets show a consistent trend, whereas mitochondrial mass and ATP5B and ATP5G proteins in IF1 overexpressed islets did not change. In cultured INS-1 β-cells, the mitochondrial network appears to have a more significant inversed relationship with IF1. Since IF1 plays a role in facilitating mitophagy^12-14^ IF1-related mitophagy for quality control and clearance of mitochondria may play a role in the mitochondrial mass and network alterations. Additionally, mitochondrial dynamic and biogenesis changes following a long-term loss- and gain-of-function in β-cells could also contribute to the mitochondrial mass and network changes. With similar reasoning, the relatively impaired energy production in IF1 OE β-cells may lead to increased vesicophagy and reduced insulin granules with the dense core of insulin contents^50^.

In summary, the present studies demonstrate that IF1 represses mitochondrial energetics, leading to impaired β-cell function and insulin release. Inhibiting IF1 in islet β-cells may serve as a novel therapeutic strategy to improve insulin storage and secretion via upregulating cellular respiration and energy production.

## Data Availability

The data that support the findings of this study are available from the corresponding author upon reasonable request.
